# EPHA2 Polymorphisms and Age-Related Cataract in India

**DOI:** 10.1371/journal.pone.0033001

**Published:** 2012-03-08

**Authors:** Periasamy Sundaresan, Ravilla D. Ravindran, Praveen Vashist, Ashwini Shanker, Dorothea Nitsch, Badrinath Talwar, Giovanni Maraini, Monica Camparini, Bareng Aletta S. Nonyane, Liam Smeeth, Usha Chakravarthy, James F. Hejtmancik, Astrid E. Fletcher

**Affiliations:** 1 Department of Genetics, Dr. G. Venkataswamy Eye Research Institute, Aravind Medical Research Foundation, Aravind Eye Hospital, Madurai, Tamil Nadu, India; 2 Aravind Eye Hospital, Pondicherry, India; 3 Dr. Rajendra Prasad Centre for Ophthalmic Sciences, All India Institute of Medical Sciences, New Delhi, India; 4 Faculty of Epidemiology and Population Health, London School of Hygiene and Tropical Medicine, London, United Kingdom; 5 Dipartimento di Scienze Otorino-Odonto-Oftalmologiche e Cervico Facciali, Sezione di Oftalmologia, Università degli Studi di Parma, Parma, Italy; 6 School of Medicine, Dentistry and Biomedical Sciences, Centre for Vision and Vascular Science, Queen's University Belfast, United Kingdom; 7 Section on Ophthalmic Molecular Genetics, National Eye Institute, Bethesda, Maryland, United States of America; University of Durham, United Kingdom

## Abstract

**Objective:**

We investigated whether previously reported single nucleotide polymorphisms (SNPs) of *EPHA2* in European studies are associated with cataract in India.

**Methods:**

We carried out a population-based genetic association study. We enumerated randomly sampled villages in two areas of north and south India to identify people aged 40 and over. Participants attended a clinical examination including lens photography and provided a blood sample for genotyping. Lens images were graded by the Lens Opacification Classification System (LOCS III). Cataract was defined as a LOCS III grade of nuclear ≥4, cortical ≥3, posterior sub-capsular (PSC) ≥2, or dense opacities or aphakia/pseudophakia in either eye. We genotyped SNPs rs3754334, rs7543472 and rs11260867 on genomic DNA extracted from peripheral blood leukocytes using TaqMan assays in an ABI 7900 real-time PCR. We used logistic regression with robust standard errors to examine the association between cataract and the EPHA2 SNPs, adjusting for age, sex and location.

**Results:**

7418 participants had data on at least one of the SNPs investigated. Genotype frequencies of controls were in Hardy-Weinberg Equilibrium (p>0.05). There was no association of rs3754334 with cataract or type of cataract. Minor allele homozygous genotypes of rs7543472 and rs11260867 compared to the major homozygote genotype were associated with cortical cataract, Odds ratio (OR) = 1.8, 95% Confidence Interval (CI) (1.1, 3.1) p = 0.03 and 2.9 (1.2, 7.1) p = 0.01 respectively, and with PSC cataract, OR = 1.5 (1.1, 2.2) p = 0.02 and 1.8 (0.9, 3.6) p = 0.07 respectively. There was no consistent association of SNPs with nuclear cataract or a combined variable of any type of cataract including operated cataract.

**Conclusions:**

Our results in the Indian population agree with previous studies of the association of EPHA2 variants with cortical cataracts. We report new findings for the association with PSC which is particularly prevalent in Indians.

## Introduction

Age-related cataract results from increasing opacification of the ocular lens eventually leading to visual loss and is a problem found in many people throughout the world as they age. Surgery with intraocular lens implantation is currently the only effective procedure. In low income countries with poor access to cataract surgery, cataract is the main cause of vision impairment and blindness [1]. In well-resourced countries cataract surgery is one of the highest health care expenditures [2]. There is evidence of a genetic component to age-related cataract. Earlier studies found that family history was a risk factor for cataract [3]–[6] while the strongest evidence came from twin studies demonstrating a heritability of 48% for nuclear cataract [7] and 59% for cortical cataract [8]. Most work on cataract genetics has focused on inherited congenital cataract and there has been limited success in identifying common genetic variants associated with age-related cataract. However recently, some genetic variants in populations of European descent have been found to be associated with cataract, primarily cortical cataract [9]–[12].

We aimed to establish if these variants confer the same risk in Indians. The prevalence of cataract in the Indian population has been found to be particularly high, even when the level of cataract surgery is taken into account, and cataract occurs at a younger age than in Western populations [13]–[15]. We focused on the Ephrin-receptor Type-A2 (*EPHA2*) on chromosome 1p. This gene was selected as a candidate because a genome-wide scan conducted within the Beaver Dam Eye study (BDES) showed linkage with cortical cataracts in that region [10]. It was the first major gene to be associated with cortical cataracts in two independent European populations [11], [12] and one study in Han Chinese [16]. In an Italian cohort, three single nucleotide polymorphisms (SNPs rs3754334, rs7543472 and rs11260867) in the 3′ region of *EPHA2* which harbours highly conserved translational control sequences for localised gene expression were found to be associated with cataracts [11]. Within this cohort, all three SNPs showed a genotypic association with cortical cataracts and rs7543472 also with nuclear cataract. The study in three European populations [12] found also that rs3754334 was associated with cortical cataract. In the Chinese study there was no association with rs3754334 [16]. We aimed to confirm the association of these three 3′ *EPHA2* SNPs with age-related cataracts in the Indian population.

## Methods

### Ethics Statement

Participants gave full informed written consent. Illiterate subjects had the information leaflet read out to them and provided a thumb impression. The study complied with the guidelines in the Declaration of Helsinki and ethics approval was received from the Indian Council for Medical Research, Research Ethics Committees of the All India Institute of Medical Sciences, Aravind Eye Hospital, London School of Hygiene and Tropical Medicine and Queen's University Belfast.

### Participants and procedures

The India age-related eye disease study (INDEYE) is a large population-based study conducted in two centers in north and south India. Our overall objectives were to examine the prevalence and risk factors for cataract and age-related macular degeneration. The study took place at two locations served by respective participating hospitals: Gurgaon district of Haryana state in north India served by Dr Rajendra Prasad Center of Ophthalmic Sciences (RPC) at the All India Institute of Medical Sciences, Delhi; and Pondicherry and Cuddalore districts of Tamil Nadu in south India served by the Aravind Eye Hospital (AEH).

A total of 59 clusters, 29 in the north and 30 in the south, were randomly selected and all people aged 40 and over were identified from household enumeration. The main INDEYE study recruited people aged 60 and over between 2004 and 2006 [15]. In 2007 to 2008 a random sample of those aged 40–59 at enumeration were invited to take part in the study with the objective of enhancing the sample for genetic studies by extending the age range to include a younger age group. Enumerators collected household and individual socio demographic and economic data (type of house, land ownership, education, occupation, caste, religion). Participants were interviewed at home by trained fieldworkers using a structured questionnaire which included tobacco use (smoking beedies (hand rolled cigarettes using Indian grown tobacco) and/or cigarettes, chewing or inhaling). Within a week of the home interview participants were brought to the base hospital for the clinical examination which included measurement of height and weight, blood sample collection, and an eye examination. A non-fasting sample of capillary blood was assessed for glucose (CBG) using a reagent strip test and reflectance meter.

Following pupillary dilation, digital slit beam images of the lens were taken using the Topcon SL-D7 Digital photo slit lamp for nuclear opacities (Topcon, Tokyo, Japan) and retroillumination images of the lens using the Neitz CT-S Cataract Screener for cortical and posterior sub-capsular opacities (Neitz Instruments Ltd., Tokyo, Japan). Lens opacities were graded according to the Lens Opacities Classification System III (LOCS III) [17] in 0.1 unit steps for each opacity up to a maximum of 6.9 for nuclear opacities, and 5.9 for cortical and PSC. The training and quality assurance of the photographers and graders and the results of the prevalence of cataracts in those aged 60 and over have been reported elsewhere [15].

### Genotyping

The 3 SNPs were genotyped on genomic DNA extracted from peripheral blood leukocytes using TaqMan assays in an ABI 7900 real-time PCR using 384 well plates. The genotyping procedure was performed according to the manufacturer's protocol. Clustering of genotypes was inspected for separation between genotypes and accuracy of genotype calls. Clustering algorithms for genotype assignment used three quality control steps: (1) all individual calls with a significant number of outlier SNPs were checked for DNA concentration and plated DNA amended accordingly, (2) a SNP pass/fail value was set for each SNP and genotyping plate, and (3) genotyping was repeated for calls that did not pass in the first run (5% of calls). All genotyped SNPs were in Hardy-Weinberg equilibrium in the controls (test p-value>0.05).

### Statistical Analysis

All analyses were done in STATA 11 (StataCorp. 2009. Stata Statistical Software: Release 11. College Station, TX: StataCorp LP.). We defined the type of cataract based on the LOCS III grade in the worse eye of: ≥4 for nuclear cataract, ≥3 for cortical cataract and ≥2 for posterior subcapsular cataract (PSC). People with any type of cataract based on these criteria or those whose images could not be graded for type of cataract because of dense opacities were included in the definition of any unoperated cataract. People with any unoperated cataract and those who were pseudophakic or aphakic in either eye were included in the definition of any cataract (i.e., operated and unoperated cataract). We used logistic regression to investigate the association between SNP genotypes and cataract or type of cataract. In all analyses the comparator group were those with no cataract or history of operated cataract (i.e. <4 for nuclear cataract, and <3 for cortical cataract and <2 for posterior subcapsular cataract, no dense opacities and no aphakia/pseudophakia). This group act as the controls in the analyses. Further models included age, sex and study centre. Age was included as a continuous variable and a quadratic effect for this was also tested. We examined the association between the homozygotes for the minor allele and heterozygotes compared to the common homozygotes. We further investigated whether including tobacco use, body mass index (BMI), diabetes, and markers of socio-economic status improved the precision of estimation. Diabetes was defined as CBG≥200 mg/dl [18]. The socio-economic index status was derived from the information collected at enumeration. These variables were included because they are indicators for potential non-random mating between social strata. All analyses took account of the cluster sampling design by estimation of robust standard errors. Out of the 5998 households from which participants were selected, there were 4144 (69.1%) with a single participant each, 1688 (28.1%) with 2 participants each, 149 (2.5%) with 3 participants each, 12 (0.2%) with 4 participants each and 3 (0.05%) with 5 participants each. Of in total 1854 households with 2 or more participants, 60.1% (1115 households), there were no biological relations, in 4.9% (90 households) there were 2 unrelated members and one person who was related to one of these, and in the remainder the biological relationships were uncertain. We ran multilevel logistic models to investigate whether household structure influenced our results.

## Results

Of 8406 people who underwent a lens examination and provided a blood sample, 619 did not consent to the genetics study. Of the remainder, 7474 could be genotyped for at least one of the EPHA2 SNPs of whom 7418 had complete information on all grades of opacity. Over half (n = 4198) had some form of cataract or had had a previous cataract operation (Table 1). Nuclear cataracts (n = 2489) were the most common type of cataract and observed in a third of participants; PSC cataracts were found in 1100 (15%), and cortical cataracts in 542 (7%). The genotype frequencies of the three SNPs by cataract status are shown in Table 2. The minor allele frequencies (MAF) in the control group were: rs3754334, 0.351 (north) and 0.344 (south) and 0.348 for both locations combined; rs7543472, 0.182 (north) and 0.229 (south) and 0.204 for both; and rs11260867, 0.091 (north) and 0.062(south) and 0.078 for both. All 3 SNPs were in Hardy Weinberg Equilibrium in the controls (rs3754334 p = 0.9, rs7543472, p = 0.8, rs11260867 p = 0.3). There was no association with rs3754334 and any cataract or any of the subtypes of cataract (Table 3). There was a weak association for an additive model for rs7543472 and any cataract (age, sex and location adjusted Odds Ratio (OR) = 1.11 (95% CI 1.08–1.22) (Table 4)). There was no evidence of association for the heterozygous genotype of rs7543472 with either cortical or PSC cataract, but there was an association for the homozygous genotype, OR = 1.83, 95% CI (1.07–3.14) with cortical cataract and with PSC, OR = 1.51, 95% CI (1.07–2.15) (Table 4). No association was seen with nuclear cataract. There was no association with any cataract or with nuclear cataract and rs11260867 (Table 5). There was evidence of an additive association with cortical cataract, OR = 1.39, 95% CI (1.07–1.80). The heterozygote and homozygote genotypes were associated with an approximately 2 and 3 fold OR respectively. For PSC cataract the ORs suggested an increased effect especially for the homozygote genotype but the 95% CIs crossed unity, OR = 1.84, 95% CI (0.96–3.57). In further analyses we found no association with any SNP and unoperated cataract of any type and no evidence of an at risk haplotype from the three SNPs for any cataract or any type of cataract. There was no improvement in precision after including a quadratic effect of age. BMI, socio-economic status, tobacco use or diabetes status or household structure did not show any evidence of confounding or effect modification.

**Table 1:** Distribution of participants by cataract status for those who have at least one of the genotyped SNPs.

	No cataract^1^	Any cataract^2^	Cortical^3^	PSC^4^	Nuclear^5^
Overall (N = 7418)	3220 (43.4%)	4198(56.6%)	542(7.3%)	1100(14.7%)	2489(33.3%)
Study location					
South, n = 3517	1495	2022	277	468	1137
North, n = 3901	1725	2176	265	642	1352
Age category					
<60, n = 2249	1763	486	77	140	273
≥60, n = 5169	1457	3712	465	970	2216
Gender					
Male, n = 3505	1619	1886	221	489	1117
Female, n = 3913	1601	2312	321	621	1372

1 “no cataract” defined as the absence of all of the following on LOCS III: cortical≥3, PSC≥2, nuclear≥4, dense opacities, operated cataract.

2 defined as any of the following on LOCS III: cortical≥3, PSC≥2, nuclear≥4, dense opacities, operated cataract.

3 LOCS III: cortical≥3.

4 LOCS III: posterior subcapsular (PSC)≥2.

5 LOCS III: nuclear ≥4.

**Table 2:** EPHA2 genotype distribution by phenotype.

	No cataract^1^	Any cataract^2^	Cortical^3^	PSC^4^	Nuclear^5^
	N (%)	N (%)	N (%)	N (%)	N (%)
**rs3754334**	3207	4195	542	1109	2487
CC	1362(42.5)	1736(41.4)	213 (39.3)	453(40.9)	1049 (42.2)
CT	1460(45.5)	1876(44.7)	262 (48.3)	502(45.3)	1106 (44.5)
TT	385(12.0)	583(13.9)	67 (12.4)	154(13.9)	332 (13.4)
**rs7543472**	3210	4190	541	1109	2485
CC	2028(63.2)	2569(61.3)	336(62.1)	690(62.2)	1544(62.1)
CT	1054(32.8)	1419(33.9)	173(32.0)	360(32.5)	831(33.4)
TT	128(4.0)	202(4.8)	32(5.9)	59(5.3)	110(4.4)
**rs11260867**	3197	4176	538	1104	2474
GG	2725 (85.2)	3527 (84.5)	445 (82.7)	924 (83.7)	2083 (84.2)
GC	448 (14.1)	623 (14.9)	86 (16.0)	169 (15.3)	379 (15.3)
CC	24 (0.7)	25 (0,6)	7 (1.3)	11 (1.0)	12 (0.5)

1 “no cataract” defined as the absence of all of the following on LOCS III: cortical≥3, PSC≥2, nuclear≥4, dense opacities, operated cataract.

2 defined as any of the following on LOCS III: cortical≥3, PSC≥2, nuclear≥4, dense opacities, operated cataract.

3 LOCS III: cortical≥3.

4 LOCS III: posterior subcapsular (PSC)≥2.

5 LOCS III: nuclear≥4.

**Table 3:** Associations between cataract and rs3754334.

Any cataract^1^ (N = 7402)	TC vs. CC	TT vs. CC	Additive model
Unadjusted	1.01(0.91–1.11) p = 0.9	1.19(1.02–1.39) p = 0.03	1.07(0.99–1.15) p = 0.08
Age, sex and locationAdjusted	0.98(0.86–1.13) p = 0.7	1.08(0.88–1.32) p = 0.5	1.02(0.93–1.12) p = 0.6
**Cortical Cataract** 2 **(N = 3749)**	TC vs. CC	TT vs. CC	Additive model
Unadjusted	1.15(0.96–1.38) p = 0.1	1.11(0.81–1.53) p = 0.5	1.08(0.94–1.24) p = 0.3
Age, sex and locationAdjusted	1.18(0.97–1.43) p = 0.2	0.94(0.67–1.34) p = 0.7	1.03(0.89–1.19) p = 0.7
**PSC Cataract** 3 **(N = 4316)**	TC vs. CC	TT vs. CC	Additive model
Unadjusted	1.03(0.87–1.22) p = 0.7	1.20(0.97–1.50) p = 0.1	1.08(0.97–1.21) p = 0.2
Age, sex and locationAdjusted	1.05(0.84–1.31) p = 0.7	1.10(0.84–1.46) p = 0.5	1.05(0.92–1.20) p = 0.5
**Nuclear Cataract** 4 **(N = 5694)**	TC vs. CC	TT vs. CC	Additive model
Unadjusted	0.98(0.87–1.11) p = 0.8	1.12(0.95–1.32) p = 0.2	1.04(0.96–1.12) p = 0.4
Age, sex and locationAdjusted	0.97(0.83–1.14) p = 0.7	1.03(0.81–1.30) p = 0.8	1.00(0.90–1.12) p = 0.9

1 defined as any of the following on LOCS III: cortical≥3, PSC≥2, nuclear≥4, dense opacities, operated cataract.

2 LOCS III: cortical≥3.

3 LOCS III: posterior subcapsular (PSC)≥2.

4 LOCS III: nuclear≥4.

**Table 4:** Associations between cataract and rs7543472.

Any cataract^1^ (N = 7400)	TC vs. CC	TT vs. CC	Additive model
Unadjusted	1.06(0.99–1.15) p = 0.1	1.24(0.97–1.60) p = 0.08	1.08(1.01–1.17) p = 0.03
Age, sex and location adjusted	1.09(0.97–1.22) p = 0.1	1.30(0.97–1.75) p = 0.08	1.11(1.08–1.22) p = 0.03
**Cortical cataract** 2 **(N = 3751)**	TC vs. CC	TT vs. CC	Additive model
Unadjusted	0.99(0.85–1.15) p = 0.9	1.51(0.97–2.34) p = 0.07	1.09(0.94–1.27) p = 0.2
Age, sex and location adjusted	1.07(0.89–1.30) p = 0.5	1.83(1.07–3.14) p = 0.03	1.19(1.00–1.43) p = 0.05
**PSC cataract** 3 **(N = 4319)**	TC vs. CC	TT vs. CC	Additive model
Unadjusted	1.00(0.88–1.14) p = 0.9	1.35(0.98–1.87) p = 0.07	1.07(0.96–1.19) p = 0.2
Age, sex and location adjusted	1.07(0.90–1.28) p = 0.4	1.51(1.07–2.15) p = 0.02	1.13(0.99–1.30) p = 0.07
**Nuclear cataract** 4 **(N = 5695)**	TC vs. CC	TT vs. CC	Additive model
Unadjusted	1.04(0.95–1.13) p = 0.4	1.13(0.90–1.42) p = 0.3	1.04(0.97–1.28) p = 0.2
Age, sex and location adjusted	1.08(0.94–1.24) p = 0.3	1.28(0.95–1.72) p = 0.1	1.10(0.99–1.23) p = 0.1

1 defined as any of the following on LOCS III: cortical≥3, PSC≥2, nuclear≥4, dense opacities, operated cataract.

2 LOCS III: cortical≥3.

3 LOCS III: posterior subcapsular (PSC)≥2.

4 LOCS III: nuclear≥4.

**Table 5:** Associations between cataract and rs11260867.

Any cataract^1^ (N = 7373)	CG vs GG	CC vs GG	Additive model
Unadjusted	1.07 (0.94–1.22) p = 0.3	0.80(0.50–1.29) p = 0.3	1.04 (0.93–1.17) p = 0.5
Age, sex and location adjusted	1.07 (0.91–1.25) p = 0.4	0.85 (0.50–1.46) p = 0.6	1.04 (0.90–1.20) p = 0.6
**Cortical cataract** 2 **(N = 3735)**	CG vs GG	CC vs GG	Additive model
Unadjusted	1.18(0.91–1.52) p = 0.2	1.79 (0. 87–3.67) p = 0.1	1.21 (0.95–1.54) p = 0.1
Age, sex and location adjusted	1.31 (0.99–1.74) p = 0.06	2.90 (1.17–7.14) p = 0.01	1.39 (1.07–1.80) p = 0.02
**PSC cataract** 3 **(N = 4301)**	CG vs GG	CC vs GG	
Unadjusted	1.11 (0.92–1.34) p = 0.3	1.35 (0.69–2.65) p = 0.4	1.12 (0.95–1.33) p = 0.2
Age, sex and location adjusted	1.07 (0.85–1.33) p = 0.6	1.84 (0.96–3.57) p = 0.07	1.12 (0.92–1.37) p = 0.3
**Nuclear cataract** 4 **(N = 5671)**	CG vs GG	CC vs GG	
Unadjusted	1.11 (0.96–1.28) p = 0.2	0.65(0.39–1.10) p = 0.1	1.05 (0.92–1.20) p = 0.4
Age, sex and location adjusted	1.09 (0.92–1.30) p = 0.3	0.64(0.34–1.20) p = 0.2	1.05 (0.90–1.23) p = 0.5

1 defined as any of the following on LOCS III: cortical≥3, PSC≥2, nuclear≥4, dense opacities, operated cataract.

2 LOCS III: cortical≥3.

3 LOCS III: posterior subcapsular (PSC)≥2.

4 LOCS III: nuclear≥4.

## Discussion

We found an association between the *EPHA2* SNPs rs7543472 and rs1120867 with age-related cataracts in the Indian population. For both SNPs the association was observed mainly with cortical and PSC cataract although there was an association between rs7543472 and any cataract (operated or unoperated). There was no strong evidence to support an association with nuclear cataract for either SNP. In contrast, an Italian based study reported associations with rs7543472 and cortical, nuclear or any unoperated cataract [11]. The Italian study was considerably smaller than ours and based on 213 age-related cataract cases and 104 clear lens controls. It is unlikely therefore that lack of power was the reason why we did not observe an association with nuclear cataracts of the order of magnitude reported in the Italian study. In our study the association with the rare homozygous (TT) and nuclear cataract was 1.28, 95% CI 0.95–1.72 which overlaps with the confidence intervals found for the same association in the Italian cohort (OR for TT compared to the other genotypes: 1.8, 95% CI (1.2–3.1)). The odds ratios and p values for cortical cataract were similar in the two studies; 2.1 95% CI (1.2–3.6) p = 0.01 in the Italian study and 1.8 (1.1–3.1), p = 0.03 in our study. There was a striking difference between the allele frequencies in the two studies; in our Indian population the T allele was the rare allele (frequency of 20.5% in controls) while in the Italian study T was the common allele (frequency of 77%). This suggests that while genetic variation in the region linked to rs7543472 is associated with cataract, the effect is unlikely to be caused by variation in the SNP itself. Our T allele frequency was also different to other populations reported in HapMap including that of HapMap Gujarati Indians from Texas (77%) and Utah Europeans' (76%). No other population had T frequencies as low as ours although two African population groups (Nigerian Yoruba and Kenyan Masai) the T frequency was around 50%. (http://www.ncbi.nlm.nih.gov/projects/SNP/snp_ref.cgi?rs=7543472 accessed September 21^st^ 2011).

In the Italian study the OR and p values for rs11260867 (95% CI) for cortical, nuclear or any unoperated cataract were respectively 2.3 (1.3–4.4) p = 0.007, 1.5 (0.8–2.6), p = 0.2 and 1.8 (1.0–3.0), p = 0.03 comparing the CC genotype to the combined G heterozygous and homozygous genotypes (C and G alleles for rs11260867 were switched in error in the published Appendix 1 of the Italian Study [11] J.F.Hejtmancik, personal communication).

In our study the respective odds ratio and p values were 2.9 (1.2–7.1) p = 0.01, 0.6 (0.3–1.2) p = 0.2, and 0.9 (0.5–1.5), p = 0.6 comparing the CC genotype to the GG genotype. The MAF for the C allele in our study (7.8%) was much lower than seen for Europeans in whom C is the common allele (MAF = 80.5%). http://www.ncbi.nlm.nih.gov/projects/SNP/snp_ref.cgi?rs=11260867 accessed September 21st 2011). Data were not available for Texas Gujarati Indians.

We found no association with rs3754334. In contrast, in the Italian study the ORs were around 3 for the associations with cortical, nuclear and any unoperated cataract. The MAF for the T allele (34.8) in our study was similar to that of Gujarati Indians (32.4%), and slightly higher than the Utah Europeans (28.8%) and the Italian cohort (28%) (http://www.ncbi.nlm.nih.gov/projects/SNP/snp_ref.cgi?rs=3754334,accessed September 21^st^ 2011).

A meta analysis of two population based studies, a twin study and a family based study [12] found that the association of rs3754334 varied across the studies. Only cortical cataract was investigated in the meta analysis. The results were presented by p values and estimates of effect (odds ratios) were not given. Of the four individual studies only one, the UK Twin Study, was significant, p = 0.03, possibly reflecting the more advanced cataracts in that study. A further sub-set analysis of severe cortical cataract provided stronger evidence. Other EPHA2 SNPS associated with cortical cataract were rs7584209, rs3768293, rs6603867, rs667816 and rs3754334. Three haplotypes were identified which between them accounted for 8% of the total variation in cortical cataract score. These SNPs were further investigated in an association study of cortical cataracts in Han Chinese [16]. Of five SNPS investigated, no association was found for rs3754334, rs3768293 or rs707455; an association was found for rs7548209 with reduced risk conferred by the C allele in contrast to an increased risk with the C allele observed in the European studies [12]. Additionally the G allele of rs477558 was associated with a lower risk.

The lack of association with nuclear cataracts in our study and the positive association with cortical cataracts is in agreement with studies in mice showing a high incidence of cortical cataract in *EPHA2* knockout mice, greater expression of EPHA2 protein in cortical lens fiber cells compared to nuclear fiber cells [12].

We also found associations with PSC cataract and rs7543472 while for rs11260867 the association was borderline. No previous studies have investigated associations between PSC and *EPHA2* genotypes, possibly because of the much lower prevalence of PSC in western populations. In contrast in our study PSC cataract was more common than cortical cataract, a finding we have described previously [15]. While this marked difference in the prevalence of PSC between India and high income countries may be partly explained by the early presentation of people with PSC to ophthalmologists in high income countries and the subsequent removal of the lens, it may also reflect genetic and environmental factors in India.

The strengths of our study are that it is large and population-based with high quality cataract grading. Participants were drawn from a random sample of small towns and villages in the north and south of the country. The relatively high prevalence of PSC cataract allowed us to investigate associations with this type of cataract. We were not able to totally exclude possible effects of population stratification or cryptic relatedness. However, for 88% of all households, participants were unrelated. Analysis taking account of household structure did not alter the results.

In summary, we have confirmed previously reported associations of two candidate SNPs (rs7543472 and rs1120867) within the *EPHA2* gene with cortical cataracts in an Indian population. The strength and magnitude of association, as indicated by the p values and odds ratios, were similar to those reported in an Italian study [11] with an approximately 2–3 fold association with the homozygous risk genotypes. We have further extended the evidence by showing associations with PSC cataract. The low frequencies of the homozygous genotypes in our population (4% and 0.7% for rs7543472 and rs11260867 respectively) and the magnitude of the odds ratios (around 2 and 3) suggest that these SNPs do not explain the high prevalence of cataract observed in India. Despite the large size of our study we were limited in our conclusions by the rarity of the risk genotypes. The strength of evidence, as assessed by p values, was weak although comparable to p values in other studies of EPHA2 SNPs [11], [12], [16]. We did not adjust for multiple comparisons because we investigated *a priori* hypotheses of associations with the three SNPs and because adjustment for highly correlated outcomes may be inappropriately conservative. The strongest evidence in the literature is for an association with cortical cataract and EPHA2 SNPs. Our results agree with this association.

Although the associations are in a similar direction for the same alleles, the differences in allele frequencies for the SNPs tested point to differences in genetic architecture in the EPHA region between Indians and other populations. Future studies need to investigate the genetic architecture of the *EPHA2* 3′ region in more detail among Indians to help identify possible common functional variants which may influence gene expression in the eye.
